# A Bimolecular Fluorescence Complementation Tool for Identification of Protein-Protein Interactions in *Candida albicans*

**DOI:** 10.1534/g3.117.300149

**Published:** 2017-08-31

**Authors:** Ana Subotić, Erwin Swinnen, Liesbeth Demuyser, Herlinde De Keersmaecker, Hideaki Mizuno, Hélène Tournu, Patrick Van Dijck

**Affiliations:** * VIB-KU Leuven Center for Microbiology, KU Leuven, 3001 Leuven, Belgium; † Laboratory of Molecular Cell Biology, Institute of Botany and Microbiology, KU Leuven, 3001 Leuven, Belgium; ‡ Laboratory of Functional Biology, Institute of Botany and Microbiology, KU Leuven, 3001 Leuven, Belgium; § Laboratory of Biomolecular Network Dynamics, Biochemistry, Molecular and Structural Biology Section, Department of Chemistry, KU Leuven, 3001 Leuven, Belgium

**Keywords:** BiFC, *Candida albicans*, protein-protein interactions

## Abstract

Investigation of protein-protein interactions (PPI) in *Candida albicans* is essential for understanding the regulation of the signal transduction network that triggers its pathogenic lifestyle. Unique features of *C. albicans*, such as its alternative codon usage and incomplete meiosis, have enforced the optimization of standard genetic methods as well as development of novel approaches. Since the existing methods for detection of PPI are limited for direct visualization of the interacting complex *in vivo*, we have established a bimolecular fluorescence complementation (BiFC) assay in *C. albicans*, a powerful technique for studying PPI. We have developed an optimized set of plasmids that allows for N- and C-terminal tagging of proteins with split yeast-enhanced monomeric Venus fragments, so that all eight combinations of fusion orientations can be analyzed. With the use of our BiFC assay we demonstrate three interaction complexes *in vivo*, which were also confirmed by two-hybrid analysis. Our *Candida*-optimized BiFC assay represents a useful molecular tool for PPI studies and shows great promise in expanding our knowledge of molecular mechanisms of protein functions.

*Candida albicans* is the most prevalent opportunistic fungal pathogen in the human population. As a commensal organism, it is part of the normal human microbiota but can also lead to infections, ranging from superficial to systemic and often lethal (Poulain 2015). Unlike the model yeast *Saccharomyces cerevisiae*, *C. albicans* has an alternative genetic code and incomplete sexual cycle, which hinder the classical genetic approaches (Santos and Tuite 1995; Noble and Johnson 2007). Hence, investigation of pathogenesis-related underlying processes, which are mediated by protein-protein interactions (PPI), is restricted to a limited number of genetic tools available in *C. albicans*. Two current methods suitable for detecting binary interactions are the *Candida* two-hybrid system (Stynen *et al.* 2010) and the vesicle capture interaction assay (Boysen *et al.* 2009). However, both genetic methods require specific localization of the proteins of interest in the cell. Another alternative approach described for detection of PPI in *C. albicans* is the “expanded genetic code” method (Palzer *et al.* 2013). In this method, a synthetic photo-cross-linker amino acid is genetically incorporated in a protein of interest to covalently capture its binding partner. It allows structural and functional analyses of PPI; however, the success of the assay depends on the incorporation efficiency of the unnatural amino acid (Wang *et al.* 2009). Since these methods either fail or exhibit limitations in visualizing the native localization of the interacting proteins, as well as testing interactions of membrane proteins *in vivo*, we sought to optimize the use of bimolecular fluorescence complementation (BiFC) in *C. albicans*. BiFC is a method based on the reassembly of a fluorescent protein as a consequence of its two complementary N- and C-terminal fragments being brought together by a pair of interacting proteins (Kerppola 2009). Consequently, the interaction complex can be directly visualized at its native subcellular location while allowing convenient quantitative analysis of the interaction by the fluorescence signal intensity. Over the last decade, the BiFC assay has been widely used for proteomic interaction studies in mammalian cells, plants, and the model yeast *S. cerevisiae* (Hu *et al.* 2002; Walter *et al.* 2004; Sung *et al.* 2013). It is of particular interest in host-pathogen interactions in plants (Lee and Gelvin 2014), yet remains to be validated in fungal pathogens.

Here we describe the development of a set of *C. albicans*-optimized BiFC plasmids for N- or C-terminal tagging of proteins with yeast-enhanced monomeric Venus (yEmVenus) fragments. To confirm the reliability of the assay, we have tested known interacting proteins, such as Snf4-Kis1, involved in carbohydrate metabolism, and components of the cAMP-dependent protein kinase A (PKA) pathway of *C. albicans*. We show the interaction of the upstream PKA receptor module Gpr1-Gpa2, and interaction of the PKA subunits Bcy1-Tpk1 and Bcy1-Tpk2. Together with complementary tools, our *Candida*-optimized BiFC assay shows immense potential in providing new insights into molecular mechanisms of PPI and signaling in *C. albicans*.

## Materials and Methods

### Strains

*C. albicans* strains used in this study are detailed in Table 1. All the BiFC strains were generated in the SN152 strain (Noble and Johnson 2005). All the two-hybrid assays were performed using the previously described SC2H3 strain (Stynen *et al.* 2010). All strains were regularly replicated from −80° on to yeast extract peptone dextrose (YPD) plates (1% yeast extract, 2% peptone, 2% glucose 1, 5 % agar). Precultures were grown overnight in 3 ml YPD and washed in Milli-Q water prior to use. For two-hybrid assays, cells were plated on synthetic complete (SC) medium (0.17% Bacto-yeast nitrogen base, 0.5% ammonium sulfate, 2% glucose) lacking appropriate amino acids (US Biologicals). The pH for solid SC media was adjusted to 6.5 and to 5.5 for liquid media. Agar plates were made with 1.5% of agar. Strains were cultured at 30°, unless stated otherwise.

**Table 1 C.** *albicans* strains

Strain	Genotype	Source
SN152	*arg4Δ/arg4Δ leu2Δ/leu2Δ his1Δ/his1Δ URA3/ura3Δ*:: *imm434 IRO1/ iro1*::*imm434*	Noble and Johnson (2005)
SC2H3	SN152 *5xLexAOp-ADH1b/HIS1 5xLexAOp-ADH1b/lacZ*	Stynen *et al.* (2010)
SC5314	Wild type	Gillum *et al.* (1984)

### Construction of plasmids

#### PCR cloning and templates:

All PCR-based cloning reactions were done using the Q5 polymerase from NEB (M0491S). For cloning of yEmVenus constructs, a full-length yEmVenus template was used, created by mutating the *C. albicans* codon-optimized yEVenus sequence [from plasmid pKT103, (Sheff and Thorn 2004)] at amino acid position 206, changing the alanine codon to a lysine codon. Cloning of the mCherry fluorophore sequence was done using the *C. albicans* codon-optimized template pMG2254, described in (Gerami-Nejad *et al.* 2009). To clone *C. albicans* genes of interest, genomic DNA was prepared from the SC5314 strain (Gillum *et al.* 1984), and used as the template in our PCR reactions. All generated plasmids are listed in Supplemental Material, Table S1; all primers used are listed in Table S2.

#### Construction of the BiFC plasmids:

**Figure 1:**
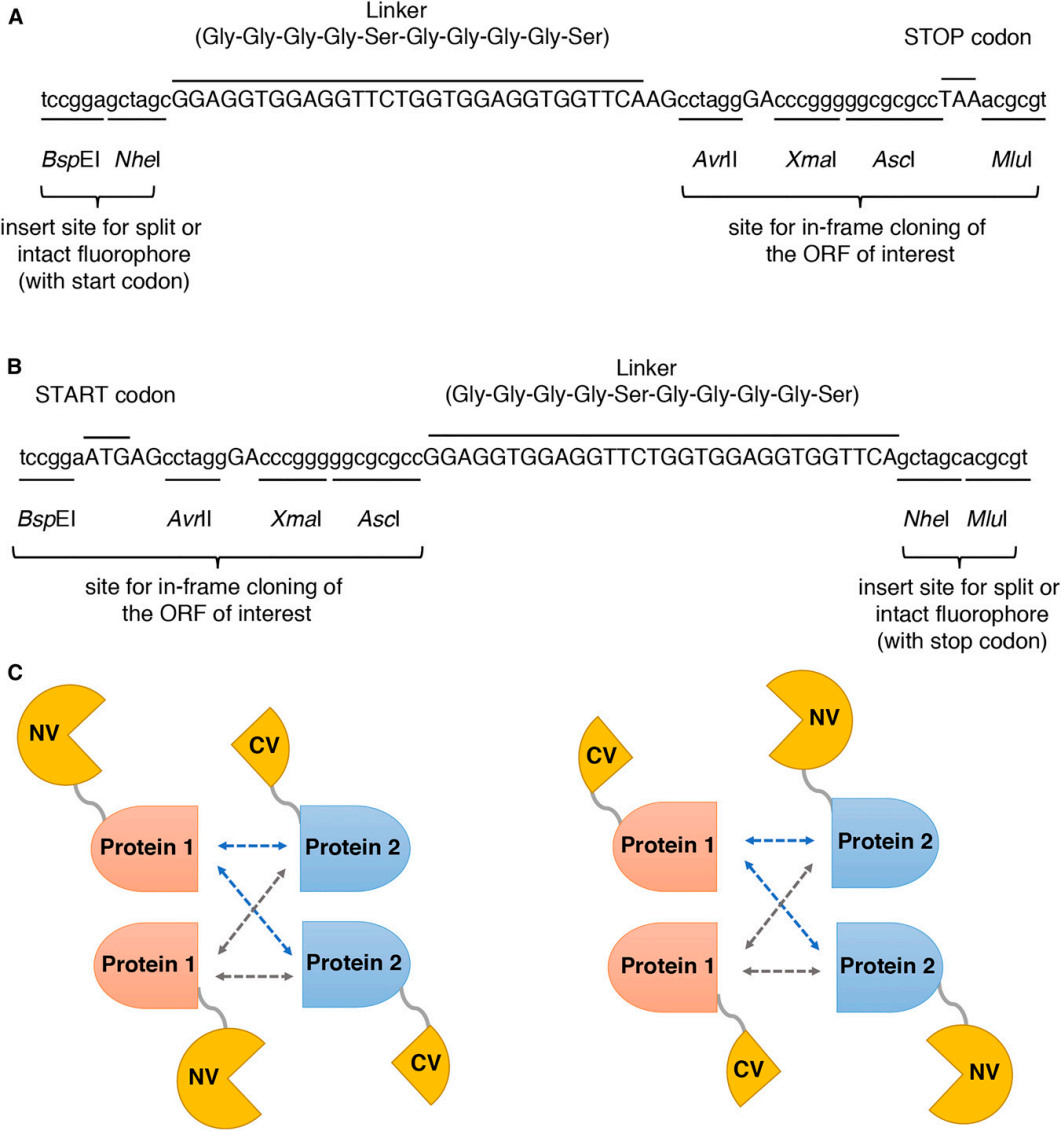
Structure of the MCS in backbone vectors for the BiFC plasmids. (A) MCS for the generation of N-terminal fusions of the fluorophore to the genes of interest. The fluorophore part is cloned in the *Bsp*EI and *Nhe*I sites on the left. Genes of interest are cloned using the *Avr*II-*Xma*I-*Asc*I-*Mlu*I cloning site on the right, separating them from the fluorophore part by the indicated linker sequence. (B) MCS for the generation of C-terminal fusions of the fluorophore to the genes of interest. The fluorophore part is cloned in the *Nhe*I and *Mlu*I sites on the right. Genes of interest are cloned using the *Bsp*EI-*Avr*II-*Xma*I-*Asc*I cloning site on the left, separating them from the fluorophore part by the indicated linker sequence. (C) Schematic representation of all possible BiFC fusion orientations with either the N-terminal (NV) or C-terminal (CV) part of yEmVenus.

The BiFC plasmids were constructed from the *C. albicans* two-hybrid vectors pC2HB and pC2HP (Stynen *et al.* 2010). In the first step, the two-hybrid specific inserts of pC2HB (NLS-LexA-HA) and pC2HP (NLS-VP16-FLAG) were cut out with *Bsp*EI and *Mlu*I, and new multiple cloning sites (MCS) were inserted by ligation with synthetic dsDNA fragments. To create the 2015-N and 2016-N vectors for N-terminal tagging of a gene of interest, the insert was formed by hybridizing the primers B-7412 and B-7413. In these vectors, the fluorophore part could be cloned into the *Bsp*EI and *Nhe*I sites, and separated by a linker sequence from the MCS (*Avr*II-*Xma*I-*Asc*I-*Mlu*I) for the gene of interest (Figure 1A). To create the 2015-C and 2016-C vectors for C-terminal tagging of a gene of interest, the synthetic dsDNA insert was formed using the primers B-7414 and B-7415. In these vectors, a slightly different MCS for the gene of interest (*Bsp*EI-*Avr*II-*Xma*I-*Asc*I) was separated by the same linker from the fluorophore part, which could then be cloned into the *Nhe*I and *Mlu*I sites (Figure 1B). Backbone vectors with the new MCS were designated as 2015-N, 2015-C, 2016-N, and 2016-C (Table 2).

**Table 2:** Backbone plasmids

Plasmid name	Genotype	Marker	Description	Source
pC2HB	—	*CmLEU2*	Bait plasmid for *C. albicans* two-hybrid assay	Stynen *et al.* (2010)
pC2HP	—	*CdARG4*	Prey plasmid for *C. albicans* two-hybrid assay	Stynen *et al.* (2010)
2015-N	—	*CmLEU2*	Backbone vector for generation of BiFC plasmids	This study
2015-C	—	*CmLEU2*	Backbone vector for generation of BiFC plasmids	This study
2016-N	—	*CdARG4*	Backbone vector for generation of BiFC plasmids	This study
2016-C	—	*CdARG4*	backbone vector for generation of BiFC plasmids	This study
BiFC1	*MET3pr*-yEmVenus (1–172)	*CmLEU2*	BiFC plasmid for N-terminal tagging of genes with yEmVenus (aa 1–172)	This study
BiFC2	*MET3pr*-yEmVenus (1–172)	*CmLEU2*	BiFC plasmid for C-terminal tagging of genes with yEmVenus (aa 1–172)	This study
BiFC3	*MET3pr*-yEmVenus (173–238)	*CdARG4*	BiFC plasmid for N-terminal tagging of genes with yEmVenus (aa 173–238)	This study
BiFC4	*MET3pr*-yEmVenus (173–238)	*CdARG4*	BiFC plasmid for C-terminal tagging of genes with yEmVenus (aa 173–238)	This study
2015-C-mCherry	*MET3pr*-mCherry	*CmLEU2*	Plasmid for C-terminal tagging of genes with mCherry	This study
2016-C-yEmVenus	*MET3pr*-yEmVenus	*CdARG4*	Plasmid for C-terminal tagging of genes with yEmVenus	This study

In the next step, the fluorophore parts were cloned into the new backbone vectors to generate the final BiFC plasmids. The large part of the yEmVenus (encoding amino acids 1–172) was cloned into both *CmLEU2* vector backbones, 2015-N and 2015-C. To generate the BiFC1 plasmid, yEmVenus (aa 1–172) was amplified with primers B-7567 and B-7568 and cloned into the 2015-N vector at the *Bsp*EI and *Nhe*I sites (Figure 1A). The BiFC2 plasmid was created by cloning yEmVenus (aa 1–172), amplified with primers B-7569 and B-7570, into the 2015-C vector at the *Nhe*I and *Mlu*I sites (Figure 1B).

The smaller complementing part of the yEmVenus fluorophore (encoding amino acids 173–238) was cloned into the *CdARG4* vector backbones, 2016-N and 2016-C. To generate the BiFC3 plasmid, yEmVenus (aa 173–238) was amplified with primers B-7571 and B-7572 and cloned into the 2016-N vector at the *Bsp*EI and *Nhe*I sites (Figure 1A). The BiFC4 plasmid was generated by cloning yEmVenus (aa 173–238), amplified with primers B-7573 and B-7574, into the 2016-C vector at the *Nhe*I and *Mlu*I sites (Figure 1B).

The completed four BiFC plasmids (Figure S1) allowed generation of all the possible BiFC fusion orientations (Figure 1C). All constructs were expressed from the *C. albicans MET3* promoter, and the *C. albicans ACT1* terminator was used in all vectors. As such, expression was repressed by the presence of methionine and/or cysteine (Care *et al.* 1999). Expression was induced by growing the cells in medium lacking both methionine and cysteine.

#### Construction of plasmids for (co)localization studies:

To study the localization of proteins, full-length yEmVenus was cloned into the 2015-N and 2016-N vectors using primers B-7567 + B-7572 for N-terminal fusions, and into the 2015-C and 2016-C vectors using primers B-7569 + B-7574 for C-terminal fusions. In addition, similar vectors were made with full-length mCherry, using primers B-7579 and B-7584 to generate vectors for N-terminal fusions, and primers B-7581 and B-7586 for C-terminal fusions. Although not all backbone vectors were used in this study, the complete set of plasmids for N- and C-terminal tagging of genes for localization studies are available on request.

#### Cloning of genes of interest into the BiFC and two-hybrid plasmids:

In general, the genes of interest (or fragments thereof) were PCR-amplified and cloned into the BiFC plasmids, using In-Fusion cloning (Clontech). For each gene, two sets of primers were used, to discriminate between cloning into the BiFC1 and BiFC3 vectors, generating N-terminal fusions (here, genes were cloned with their stop codons), and cloning into the BiFC2 and BiFC4 vectors, generating C-terminal fusions (here, the genes were cloned without their stop codons).

All BiFC plasmids were confirmed by sequencing. The complete list of plasmids and primers used in this study is given in Table S1 and Table S2, respectively.

### Candida two-hybrid analysis

For the two-hybrid analysis, genes of interest were cloned by the classical restriction-ligation method into the prey and bait vectors, pC2HP and pC2HB, containing the *C. dubliniensis ARG4* or *C. maltosa LEU2* marker for selection on growth media lacking arginine or leucine, respectively (Stynen *et al.* 2010). Constructs expressing the full-length *SNF4* and *KIS1* genes were retrieved from Stynen *et al.* 2010, and the pC2HP vector expressing the truncated *KIS1* (lacking its C-terminal amino acids 327–412) was made in this study. All two-hybrid plasmids were confirmed by sequencing. Both prey and bait vectors were transformed into the SC2H3 reporter strain, which is auxotrophic for arginine, leucine, and histidine. As with the BiFC constructs, expression of the two-hybrid constructs is controlled by a *MET3* repressible promoter, and thus induced by growing the strains in media lacking both methionine and cysteine (Care *et al.* 1999). The SC2H3 strain offers two reporter systems for PPI readout, a colorimetric reaction based on the expression of a *lacZ* gene, and growth on medium lacking histidine, due to the expression of the auxotrophic *HIS1* marker upon interaction of the proteins (Stynen *et al.* 2010). In this paper, interaction was scored by analyzing growth on media lacking methionine, cysteine, and histidine only.

### Construction of strains

All *C. albicans* transformations were performed using a lithium acetate method (Gietz *et al.* 1995). Prior to transformation, all plasmids were linearized with the *Not*I restriction enzyme for integration. BiFC1, BiFC2, and bait pC2HB plasmids integrated between loci *XOG1* and *HOL1* on chromosome 1, while the BiFC3, BiFC4, and prey pC2HP plasmids integrated between *RXT3* and *ORF19*.*3569* on chromosome 2 (Stynen *et al.* 2010). Integration of plasmids was verified by PCR diagnosis.

### Microplate reader measurements

The H1 Synergy microplate reader (BioTek) was used for intermittent 24 hr measurements of optical density (OD) at 600 nm and fluorescence with excitation 485/20 and emission 528/20 nm filters. For these measurements, all cultures were grown overnight in YPD, washed, diluted to OD ≈ 0.1, and transferred to low-fluorescence SC –methionine –cysteine medium (2% glucose). A strain containing the backbone vectors 2015-N and 2016-N (Table 2) was used to measure cellular autofluorescence under our experimental conditions. For all the tested strains, fluorescence intensity was normalized for cell density, by calculating the ratio of relative fluorescence units over the OD_600nm_. Then, these values for the test strains were divided by the corresponding values for autofluorescence, giving a final relative fluorescence intensity due to the BiFC interaction in our test strains.

### Confocal microscopy and quantitative analysis of images

For fluorescence imaging, cells were grown overnight in YPD, washed, and transferred to low-fluorescence SC –methionine –cysteine medium supplemented with low-fluorescence YNB (Formedium) or standard SC –methionine –cysteine medium (2% glucose) until the midexponential phase when they were visualized. Imaging was performed on an Olympus FluoView FV1000 confocal laser scanning microscope equipped with UPLSAPO 60× O NA 1.35 objective lens. yEmVenus fluorescence was excited with the 515 nm laser line emitted from an argon laser, and emission was detected through a bandpass filter BA535-565. Nuclear staining was performed with NucBlue Live ReadyProbes (Thermo Fisher Scientific) according to the manufacturer’s protocol. NucBlue stain was excited with the 405 nm laser diode and detected through a bandpass filter BA430-470. mCherry fluorescence was excited with the 559 nm laser diode, and emission was detected through a bandpass filter BA575-675. Quantification of the signal was done with a home-developed MATLAB routine (version 7.11.0.584; MathWorks, Natick, MA), which is available upon request. Cells were discriminated from the background by an adaptive local threshold combined with morphological operations to fill and close the areas. Afterward, the selected areas were visually checked. The mean background intensity per pixel was calculated based on a selected area in the image without any cell present. Thereafter, the mean intensity per pixel, corrected by the mean background intensity, was calculated for each cell separately. Analysis and comparison of fluorescence intensity was done with raw images acquired with identical acquisition settings of the microscope (laser power, detector sensitivity, and pixel dwell time). For optimal display, copies of all original images and of their controls were equally adjusted for brightness, contrast, and color balance using Adobe Photoshop CS6.

### Western blot analysis

Strains were grown overnight in YPD, washed, and transferred to SC –methionine –cysteine medium (2% glucose) until the exponential phase. Cells were then harvested and washed with lysis buffer [200 mM sorbitol, 20 mM HEPES–KOH (pH 6.8), 1 mM EDTA, 50 mM potassium acetate, and protease inhibitors (Roche)]. Next, the cells were broken with glass beads using a FastPrep machine. The concentration of proteins was determined with the Pierce protein assay (Thermo Fisher Scientific), and 14 µg/well was loaded on an Invitrogen NuPAGE Novex bis-Tris gradient gel (4–12%). For detection of the BiFC constructs, a polyclonal anti-GFP antibody (GeneTex, GTX113617) was used, which recognizes both the N- and C-terminal fragments of the yEmVenus fluorescent protein. Detection of histone H3 (loading control) was done using an anti-histone H3 antibody (Abcam, ab1791). The blots were visualized using a FujiFilm LAS-4000 mini system. Quantification was carried out with ImageJ 1.51d software (National Institutes of Health). The intensity of each band was corrected for the background and normalized to the intensity of its respective loading control band.

### Staining of vacuoles

The cells were incubated with FM4-64 (*N*-(3-triethylammoniumpropyl)-4-(6-(4-(diethylamino) phenyl) hexatrienyl) pyridinium dibromide, Invitrogen) at a final concentration of 0.04 mg/ml for 30 min at 30°. After being washed, the cultures were imaged. The dye was excited with the 559 nm laser diode and emission was detected through a bandpass filter BA575-675.

### Data availability

Complete lists of the plasmids and primers used in this study are given in Table S1 and Table S2, respectively. The four BiFC plasmids (Figure S1) are available from the Belgian Coordinated Collections of Microorganisms (BCCM/LMBP plasmid collection; http://bccm.belspo.be/about/lmbp.php) with the following accession numbers: LMBP 10464 (BiFC1), LMBP 10465 (BiFC2), LMBP 10466 (BiFC3), LMBP 10467 (BiFC4). Their sequences are available as supplementary GenBank standard files (File S1, File S2, File S3, and File S4), while the sequences of all the other plasmids used in this study are available on request. Clustal Omega is a multiple sequence alignment program available online at http://www. ebi.ac.uk/Tools/msa/clustalo/. ImageJ software from the National Institutes of Health is available online at https://imagej.nih.gov/ij/. The MATLAB code is available upon request.

## Results

### Development of Candida-optimized BiFC plasmids

*Candida*-optimized BiFC plasmids were designed for tagging genes of interest, either N- or C-terminally, with split yEmVenus fragments (Table 2). Monomeric yEVenus was created by introducing the A206K mutation in the *C. albicans* codon-optimized yEVenus sequence (Sheff and Thorn 2004). Generally, monomeric fluorescent proteins display less aggregation and are nontoxic when expressed in cells (Shaner *et al.* 2005). In our BiFC system, we split the yEmVenus in the loop between the eighth and ninth β-strands of the fluorophore, as was done by others (Kodama and Hu 2012). As such, our two yEmVenus fragments were a large fragment, from amino acid 1–172, and the complementing smaller fragment, encompassing amino acids 173–238. The BiFC plasmids contained the *MET3* repressible promoter (Care *et al.* 1999) for expression of gene fusions and either the *C. dubliniensis ARG4* or *C. maltosa LEU2* marker for selection on growth media lacking arginine or leucine, respectively. As appropriate start and stop codons were incorporated into the MCS sequences (Figure 1, A and B), the fluorophore segments were expressed even in the absence of the target gene. As such these empty BiFC vectors could be used as negative controls (self-assembly of the fluorophore) for BiFC analyses.

### Proof-of-principle experiment—interaction of Snf4-Kis1 proteins

**Figure 2:**
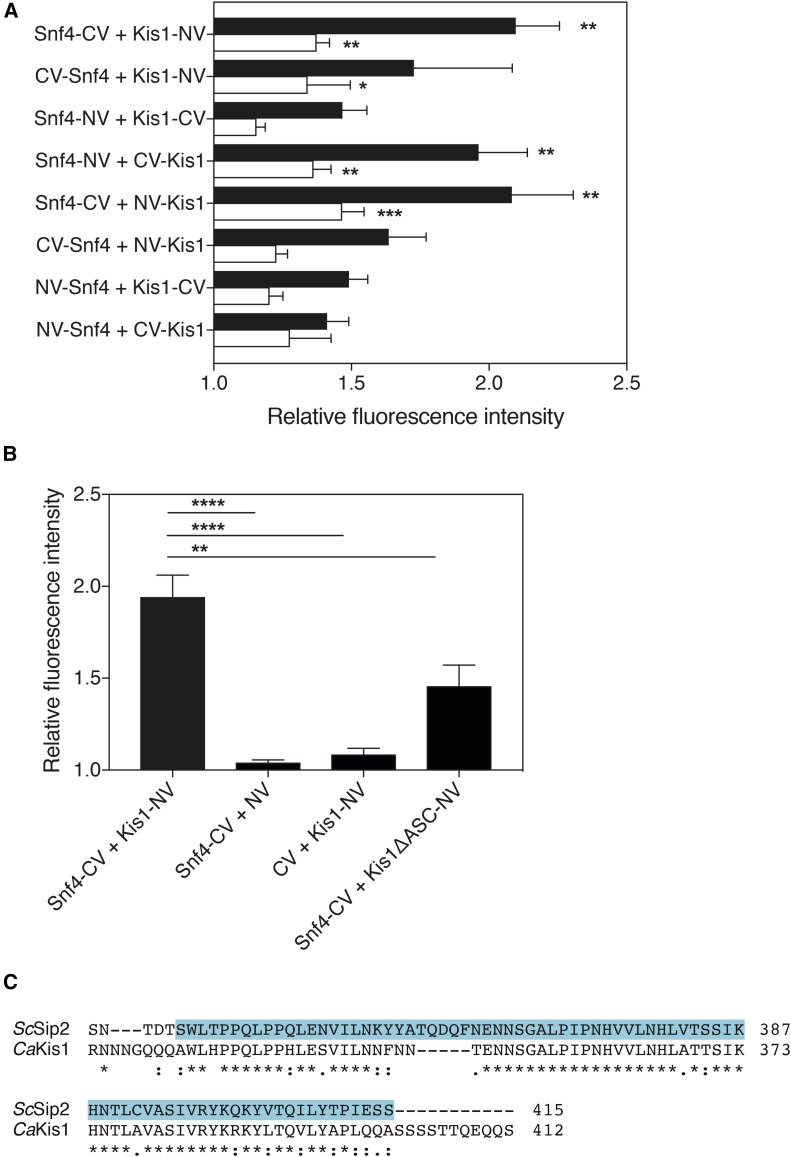
(A) Microplate reader analysis of all eight Snf4-Kis1 BiFC combinations. Quantitative analysis of Snf4-Kis1 BiFC signal in the exponential phase (black bars) and stationary phase (white bars). Relative fluorescence intensity represents the ratio of BiFC strains’ fluorescence over fluorescence of background strain containing backbone vectors. Data represent mean values ± SEM (*n* = 5). The experiment was performed twice with similar results; a representative experiment is shown. *Significant difference compared with the background strain determined in the same growth phase (two-way ANOVA, Sidak’s multiple comparisons test, * *P* ≤ 0.05, ** *P* ≤ 0.01, *** *P* ≤ 0.001). (B) Microplate reader analysis of the optimal Snf4-Kis1 BiFC combination by including the negative controls. Snf4-C-yEmVenus and Kis1-N-yEmVenus both tagged C-terminally were selected as the optimal combination. Kis1ΔASC shows significantly less fluorescence complementation with Snf4 compared to the full-length Kis1. Fluorescence was analyzed by the microplate system, in the exponential phase of growth. Relative fluorescence intensity represents the ratio of BiFC strains’ fluorescence over fluorescence of background strain containing backbone vectors. Data represent mean values ± SEM (*n* = 5). The experiment was performed twice with similar results; a representative experiment is shown. *Significant difference compared to control strains (one-way ANOVA, Dunnett’s multiple comparisons test, ** *P* ≤ 0.01, **** *P* ≤ 0.0001). (C) Alignment of the *C. albicans* Kis1 and homologous Sip2 protein in *S. cerevisiae*. The identified ScSip2 ASC domain (Jiang and Carlson 1997) is highlighted in blue. Alignment was performed using the Clustal Omega online tool.

**Figure 3:**
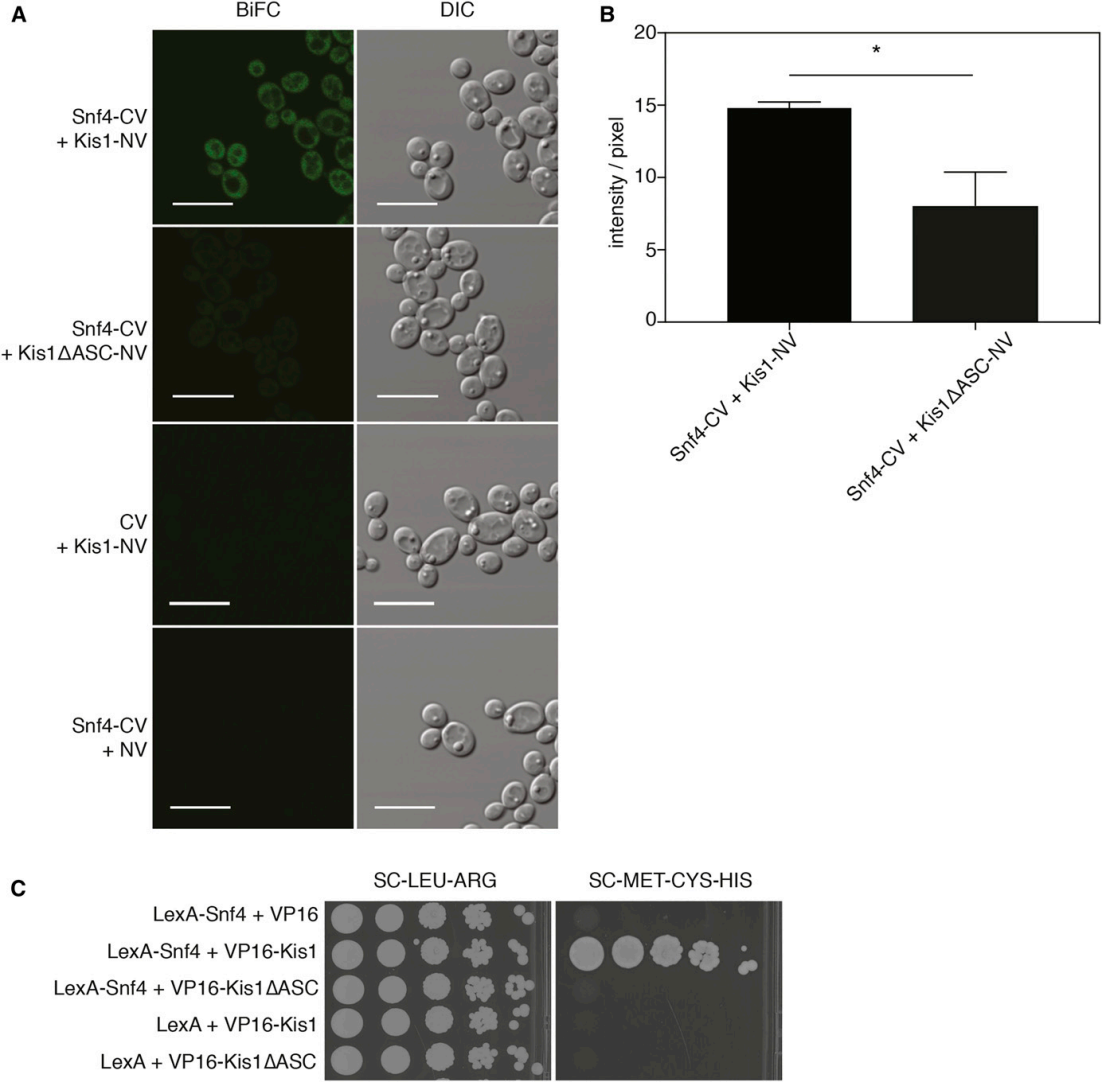
(A) Representative BiFC images showing *in vivo* interaction of Snf4-CV + Kis1-NV and control strains Snf4-CV + Kis1ΔASC-NV, CV + Kis1-NV, and Snf4-CV + NV. Bar is 10 µm. (B) Quantification of fluorescence intensity per pixel of strains Snf4-CV + Kis1-NV and Snf4-CV + Kis1ΔASC-NV. Controls could not be quantified as no signal was detected. Analysis of fluorescence intensity was done with original images using the same acquisition settings of the microscope. Data represent mean values ± SEM of (*n* = 3; ±100 cells per replicate). *Significant difference (unpaired *t*-test, * *P* ≤ 0.05). (C) Two-hybrid interaction of *C. albicans* Kis1 with Snf4 depends on the ASC domain of Kis1. Growth of all strains on SC -leucine - arginine indicates the presence of both yeast two-hybrid constructs, whereas growth on SC -methionine -cysteine -histidine demonstrates interaction between the proteins. Plates were incubated for 3 d at 30° before pictures were taken.

To validate the BiFC assay for use in *C. albicans*, we first tagged *SNF4* and *KIS1*, which encode known interacting proteins previously shown using the *Candida* two-hybrid method (Stynen *et al.* 2010). Snf4 and Kis1 belong to the Snf1 kinase complex, which is involved in the regulation of carbohydrate metabolism and growth adaptation on nonfermentable sugars in *C. albicans* (Corvey *et al.* 2005). To check for interaction, *SNF4* and *KIS1* were cloned into the BiFC plasmids and integrated in the SN152 strain. Strains expressing all eight tagging combinations were tested with the microplate reader system. The BiFC orientation expressing Snf4-CV (C-terminal tagging with the shorter C-terminal segment of the fluorophore) and Kis1-NV (C-terminal tagging with the longer N-terminal segment of the fluorophore) showed the highest fluorescence complementation compared to the background, as well as the highest reproducibility in fluorescence levels (Figure 2A). Also, a significantly higher reassembly-derived fluorescence signal, relative to the autofluorescence under the same conditions, was observed in the exponential phase than in the stationary growth phase (Figure S2). Therefore, for this BiFC orientation, appropriate negative controls were generated and the fluorescence signals were reanalyzed compared to these controls during the exponential growth phase (Figure 2B). Since both negative controls showed nearly no nonspecific complementation, we proceeded to microscopical analysis. When visualized, the two proteins strongly interacted in glucose-containing media with the fluorescence signal occurring throughout the cytoplasm (Figure 3A).

It was previously shown in *S. cerevisiae* that *Sc*Snf4 interacts with *Sc*Sip1/Sip2/Gal83 through an ASC (association with the Snf1p kinase complex) domain (Jiang and Carlson 1997). A homologous domain, aa 327–412, was also identified in the *C. albicans KIS1* gene (Figure 2C) (Corvey *et al.* 2005). When integrated in SN152, the truncated Kis1 showed significantly less fluorescence complementation with Snf4 compared to the full-length Kis1, in both the microplate system and in quantitative analysis of microscopy images (Figure 2B and Figure 3B). This loss of interaction was also confirmed using the *Candida* two-hybrid assay (Figure 3C). In addition, we assessed the protein expression levels of the full-length Kis1-NV and the Kis1ASCΔ-NV protein fusions in the Snf4-Kis1 BiFC strains. For the western blot analysis, we used an anti-GFP antibody, which recognizes both N- and C-terminal yEmVenus fragments. The results showed that the amount of the Kis1ASCΔ protein was decreased compared to the full-length Kis1-NV, indicating that the deletion of the ASC domain affects its stability (Figure S3, A and B). However, the extent to which the instability of Kis1 is a cause or consequence of its loss of interaction with the complex remains to be investigated. Overall, these results indicate that it can be important to assess the stability of fusion proteins to verify that they are present in comparable amounts and to exclude the possibility of false negative BiFC results.

### Application of BiFC on the interaction of membrane-bound Gpr1 and Gpa2

G-protein coupled receptors (GPCRs) are among the best-studied membrane sensors in eukaryotes, and the *Sc*Gpr1-Gpa2 GPCR system in *S. cerevisiae* represents the first-described glucose-sensing GPCR in eukaryotes (Kraakman *et al.* 1999). Similarly, in *C. albicans*, Gpr1 together with Gpa2 acts upstream of the cAMP-PKA pathway and is involved in morphogenesis on different solid hyphae-inducing media (Maidan *et al.* 2005). One of the main advantages of the BiFC assay over the two-hybrid system is that it enables detection and visualization of PPI involving membrane proteins. Therefore, we have employed our BiFC system for visualizing the interaction between Gpr1 and Gpa2. The physical interaction between the C-terminal region of *C. albicans* Gpr1 (aa 660–823) and Gpa2 had already been shown using the *S. cerevisiae* two-hybrid assay (Miwa *et al.* 2004). Using the BiFC system, we tested the interaction of the full-length Gpr1 and the C-tail of Gpr1 (aa 660–823) with Gpa2 in *C. albicans*. Gpr1 was only tagged C-terminally, as its N-terminus is extracellularly located. As such, only four possible tagging combinations were tested, together with their respective controls. Compared with the Snf4-Kis1 interaction, the signal of Gpr1-Gpa2 interactions was much lower, reaching the detection limit of the microplate reader. Therefore, the Gpr1-Gpa2 BiFC combinations could not be scored using the microplate reader system, and selection was performed by direct imaging of the different combinations under the microscope.

**Figure 4:**
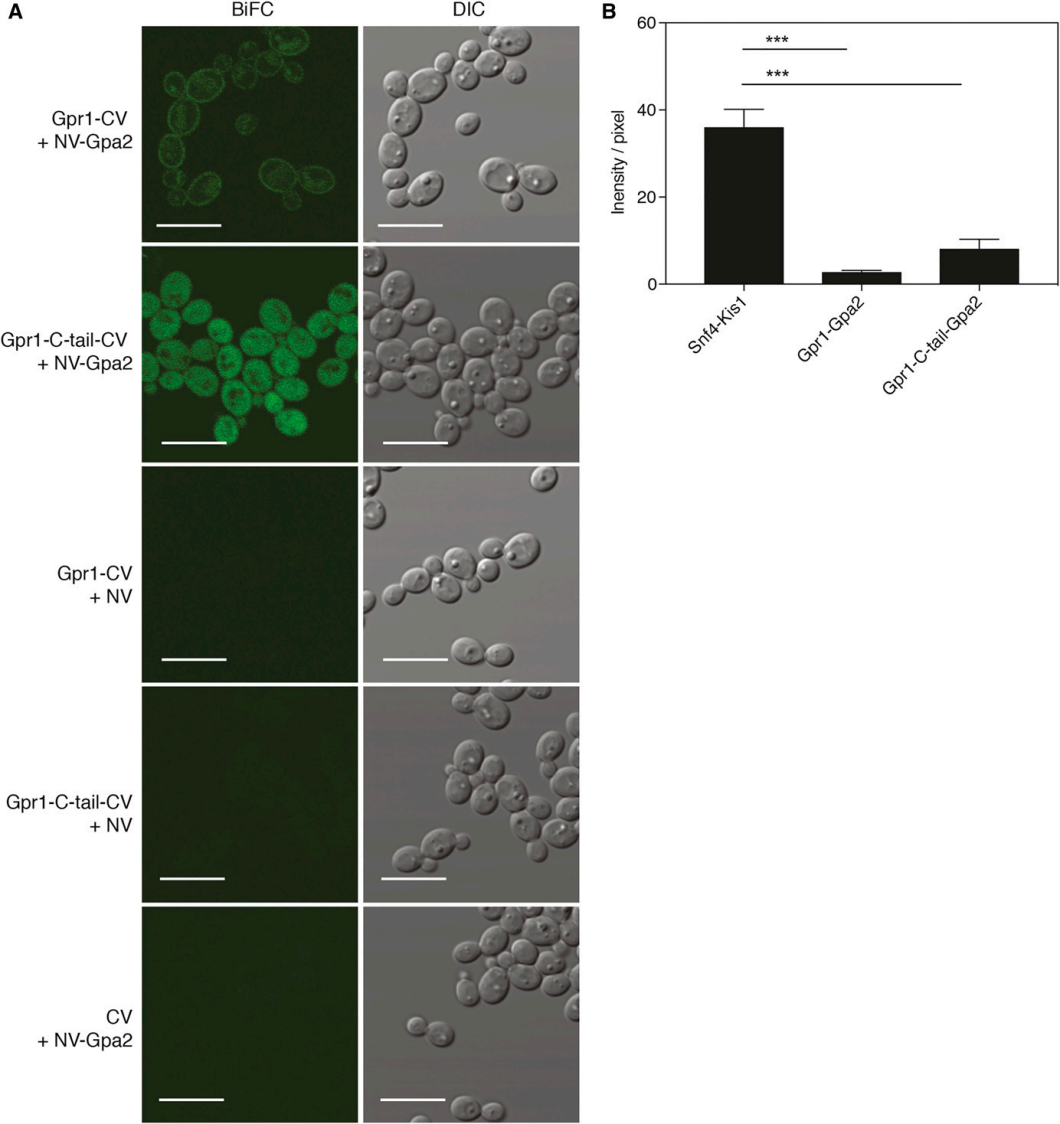
(A) Representative BiFC images showing *in vivo* interaction of Gpr1-CV + NV-Gpa2, the C-tail of Gpr1 and Gpa2, and control strains expressing Gpr1-CV + NV, Gpr1-C-tail-CV + NV, and CV + NV-Gpa2. Bar is 10 µm. (B) Quantification of fluorescence intensity per pixel of Snf4-Kis1, Gpr1-Gpa2, and Gpr1-C-tail-Gpa2 strains. Analysis of fluorescence intensity was done with original images using the same acquisition settings of the microscope. Data represent mean values ± SEM (*n* = 3; ±60 cells per replicate). *Significant difference (one-way ANOVA, Tukey’s multiple comparison test, *** *P* ≤ 0.001).

Visualization of these strains showed a membrane-localized interaction of full-length Gpr1 with Gpa2, whereas the C-tail of Gpr1 interacted with Gpa2 in the cytosol (Figure 4A). Intriguingly, for the full-length Gpr1 interaction, an additional fluorescent signal was observed, which localized to the vacuole, indicating degradation of the interacting complex. As we observed a similar pattern of membrane localization, along with a vacuolar signal, when solely expressing the Gpr1-yEmVenus from the same vector (Figure S4), it could be that the observed vacuolar targeting was due to the regular endocytic recycling process of Gpr1. Furthermore, this vacuolar signal was missing from strains showing the interaction of Gpa2 with the C-tail of Gpr1 only.

In agreement with the observed lower signal in the microplate reader system, quantification of microscopy images of Gpr1-CV + NV-Gpa2 and Gpr1-C-tail-CV + NV-Gpa2 strains confirmed a significantly lower signal compared to the Snf4-Kis1 BiFC signal (Figure 4B). Interestingly, the interaction of Gpa2 with the C-tail of Gpr1 appeared stronger than when full-length Gpr1 was used. This may be explained by the observed vacuolar targeting of specifically full-length Gpr1 upon overexpression. In addition to the low signals in the fluorescent assays, in a parallel two-hybrid analysis of all interactions, the Gpr1-C-tail-Gpa2 interaction strain showed less growth on selective medium compared to other interaction strains (Figure S5). These different interaction assays may point to a weak interaction between Gpr1 and Gpa2, compared to the Snf4-Kis1 tested in the same conditions, although it is also possible that our testing conditions were not optimal for maximal Gpr1-Gpa2 interaction. It is, thus, not clear yet to what extent this Gpr1-Gpa2 interaction is inherently weaker. Overall, these results demonstrate that the BiFC assay can detect binary complexes involving membrane-bound proteins *in vivo*.

### BiFC interaction of Bcy1-Tpk1 and Bcy1-Tpk2 subunits of PKA pathway

Visualization of the Gpr1-Gpa2 PKA receptor module led us to further characterize the interaction of the PKA subunits using BiFC in *C. albicans*. Besides being involved in growth and stress response, PKA is the key regulator of yeast-to-hyphae transition in *C. albicans* (Fuller and Rhodes 2012). In *C. albicans*, PKA consists of two catalytic subunits, Tpk1 and Tpk2, which interact and are regulated by Bcy1, the regulatory subunit. Environmental cues specific to the host niche, such as serum, CO_2_, and changes in pH and body temperature (37°), alter the intracellular level of cAMP, which binds to the Bcy1 subunits, in turn releasing the active Tpk1 and Tpk2. Subsequently, active Tpk1 and Tpk2 promote hyphae development through the activation of a downstream transcription factor, Efg1 (Stoldt *et al.* 1997; Sudbery 2011).

Remarkably, little is known about PKA activity and its regulation *in vivo* in *C. albicans*. Previous attempts to assess the localization of PKA subunits in *C. albicans* were only possible in stationary-phase cells owing to low expression of endogenously tagged subunits in exponentially growing cells (Cassola *et al.* 2004; Giacometti *et al.* 2006).

**Figure 5:**
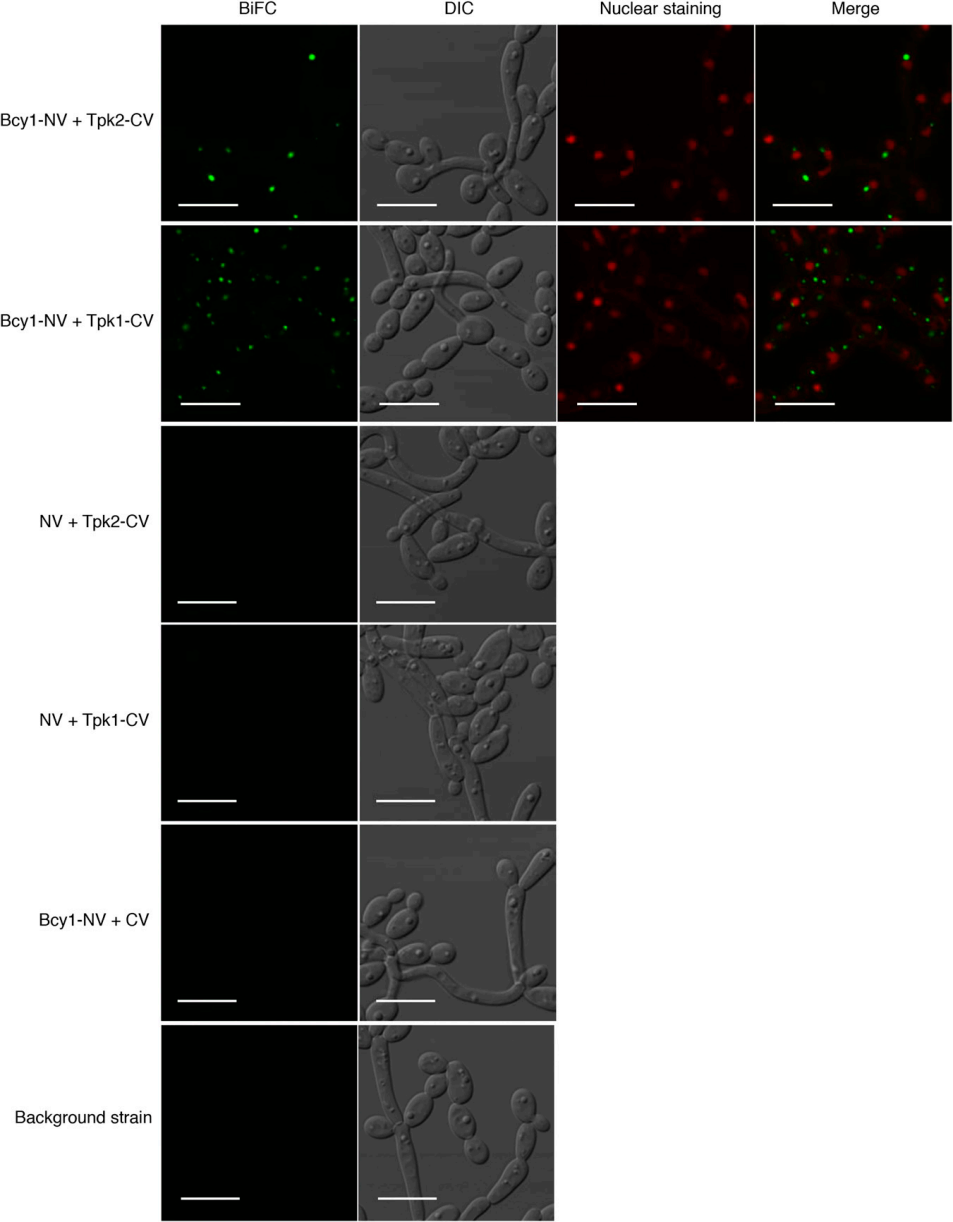
Representative BiFC images showing *in vivo* interaction of Bcy1-NV + Tpk1-CV, Bcy1-NV + Tpk2-CV with nuclear staining, controls strains expressing Bcy1-NV + CV, NV + Tpk1-CV, NV + Tpk2-CV, and the background strain. Images are taken in the exponential phase of growth in SC-Met-Cys 2% glucose at 37°. Bar is 10 µm.

Here, we applied our BiFC system to test the interaction of both catalytic subunits, Tpk1 and Tpk2, with Bcy1. Initial selection of combinations was done by the microplate reader system. However, further analysis with the microplate reader detected high nonspecific fluorescence of both Tpk1- and Tpk2-tagged negative controls. The same nonspecific signal was detected when we expressed the tagged Tpk1 or Tpk2 without the other complementary fluorophore part (data not shown), indicating that the overexpression of only catalytic subunits leads to a production of fluorescence in the media, independent of BiFC complex formation. Previously reported data showed that a hyperactive adenylate cyclase mutant leads to increased production and secretion of riboflavin (Bai *et al.* 2011), which is inherently fluorescent under the same conditions we used for detection of total fluorescence in the microplate reader system. Other unpublished results from our laboratory confirmed that overexpression of the genes encoding the catalytic Tpk subunits induces riboflavin production, which is secreted in the media (data not shown). Therefore, we have based the selection of optimal combinations on microscopy data, which proved to be stringent and most relevant in this case. Based on the microscopic analysis of the interaction strains together with their negative controls, both C-terminally tagged Bcy1 and Tpk subunits were selected as the optimal combinations. Imaging of interaction strains showed that Tpk1 and Tpk2 interact with Bcy1 in the cytoplasm and form interaction complexes observed as fluorescent foci in the exponential phase (Figure 5). Nuclear staining of these strains excluded the presence of these foci in the nucleus (Figure 5). To further examine these results, we also performed colocalization experiments with Bcy1 and Tpk subunits, tagged with full-length yEmVenus and mCherry, respectively. We could observe that both Bcy1 and Tpk2 were predominantly present in the cytoplasm and did not localize to the nucleus, whereas Tpk1 was present in both the cytoplasm and the nucleus (Figure S6). This indicates that, under our experimental conditions, there is a fraction of nuclear Tpk1, free from Bcy1-mediated inhibition, which might be involved in promoting filamentous growth. In agreement with such a Tpk1-specific role in promoting filamentation is the observation of increased Tpk1 genomic binding sites during hyphal induction using chromatin immunoprecipitation on chip in *C. albicans* (Schaekel *et al.* 2013). Also, it was shown that Tpk2 is present in the cell in higher amounts than Tpk1 (Cloutier *et al.* 2003), so the specific upregulation of Tpk1 in the nucleus might have a physiological reason. Overall, these observations indicate that the interaction of PKA subunits takes place in the cytoplasm, as shown by the BiFC signal; however, the subunits did not show inclusions that would correspond to formation of foci. Both colocalization and BiFC experiments were done by overexpressing both the Bcy1 and Tpk subunits using the same vector backbone and promoter (*MET3*). The only difference was the tagging of both subunits, either fused with a full-length yEmVenus/mCherry, or with the BiFC yEmVenus split fragments. Therefore, we can assume that the amount of overexpression is comparable in both systems. As such, the reason we observed the foci only in the BiFC strain was not an effect of stress caused by overproduction, which also occurred in the colocalization experiment. Furthermore, we observed a similar pattern of filamentation for both the colocalization and BiFC strains, as well as the control strains tested in our conditions (Figure 5 and Figure S6).

One possible explanation of the foci formation is that the interaction between Bcy1 and Tpk occurs transiently at the specific location, and this can be seen owing to the irreversible reconstitution of the BiFC complex, which stabilizes the interaction. This stabilized complex then possibly accumulates at the observed location, forming large foci. Formation of such large foci is probably not due to aggregation of the fluorophores, since we used a monomeric variant of yEVenus that is less prone to dimerize, but possibly an artificial enrichment of the interaction complex. Furthermore, no foci were observed when analyzing the Snf4-Kis1 interaction, implying that this is not a general phenomenon for cytoplasmic interaction in our BiFC system. However, further investigation of the functional and biological significance of these PKA interaction sites is necessary to fully describe their function in *C. albicans*.

## Discussion

In this study, we have generated a set of *Candida*-optimized BiFC plasmids for interaction analysis of proteins of interest. Our set of BiFC plasmids allows for N- and C-terminal tagging of proteins with split yEmVenus fragments in a way that all eight combinatorial possibilities can be analyzed.

Optimally, the BiFC assay is performed with endogenously tagged proteins rather than overexpression. However, we have opted to use a BiFC plasmid-based system, which allows for controlled expression via the *MET3* promoter, and thereby enables detection independent of native expression, which may be too low for visualization. Another advantage of this system is that it enables tagging of genes in all eight combinations. Testing all combinations allows for selection of the optimal combination of fusion proteins and avoids possible topological constraints of certain tag orientations (Miller *et al.* 2015). If desired, our BiFC plasmids for C-terminal tagging are suitable as templates for endogenous tagging as well.

Using our BiFC assay, we could visualize the cytosolic interaction of the Snf4-Kis1 pair. For this specific pair of proteins, easy quantitative analysis, using a microplate reader setup, proved to be useful for selecting the optimal interacting combination. We also tested the importance of the ASC domain of Kis1 for the interaction with Snf4. Interestingly, we found that truncation of this domain compromised the stability of Kis1. Previous research on the mammalian AMP-activated protein kinase complex has indicated that expression of all subunits was necessary for optimal stability of the entire complex, in both mammalian and yeast cells (Daniel and Carling 2002; Ye *et al.* 2014). This indicates that when subunits are not part of the complex, *e.g.*, because of loss of their ability to interact with other subunits, their stability decreases. Hence, further research is needed to show whether the ASC domain of Kis1 is necessary for the protein’s inherent stability, or whether the instability of the truncated Kis1ΔASC is a result of the reduced interaction with its partner, Snf4.

Second, we have confirmed and visualized the interaction of a membrane-bound receptor, Gpr1 and Gpa2. Full-length Gpr1 interacted with Gpa2 in the membrane, whereas the C-tail, devoid of the membrane-anchoring part, formed a complex with Gpa2 in the cytosol. Markedly, the BiFC signal of these interactions was much lower compared to Snf4-Kis1. As these differences could be due to our specific testing conditions, analyzing different environmental conditions using our BiFC system may help identify environmental cues that which influence Gpr1-Gpa2 coupling, and as such provide more information on the regulation of the PKA pathway by this GPCR. Still, these observations demonstrate that our BiFC assay is suitable for detection of both strong and weak interactions. However, a quantitative analysis using our microplate reader for selection of the optimal interacting combination was not suitable for weak BiFC signals. Therefore, depending on the detection limits of the microplate reader used, quantitative analysis of weak interactions may depend on fluorescence microscopic imaging.

Finally, besides demonstrating interaction of the upstream membrane receptor system of the cAMP-PKA pathway, Gpr1-Gpa2, our BiFC assay proved to be useful in visualizing *in vivo*, for the first time, the interaction of PKA subunits Bcy1-Tpk1 and Bcy1-Tpk2 in *C. albicans*. We could observe that, in the exponential growth phase, both Tpk1 and Tpk2 interacted with Bcy1 in the cytoplasm, forming specific localized interaction complexes. Colocalization of subunits showed that Bcy1 and Tpk2 were situated in the cytoplasm, while only Tpk1 had a nucleocytoplasmic localization, indicating that Tpk1 specifically might have a role in transcriptional regulation of filamentation in our experimental conditions. Distinct roles of the Tpk1 and Tpk2 catalytic subunits have been described not only for morphogenesis in liquid *vs.* solid media (Bockmuhl *et al.* 2001), but also in stress response and carbohydrate metabolism in *C. albicans* (Giacometti *et al.* 2009). However, in the colocalization experiment, the subunits did not show inclusions corresponding to the BiFC interaction foci. Similar to the observed BiFC foci, previous studies identified lipofuscin-like autofluorescence bodies in *C. albicans* (Diaz *et al.* 2005). Still, the occurrence of these bodies usually precedes cell death and is accompanied by evident morphological changes, which were not observed with the BiFC strains. The BiFC strains, together with the controls and the colocalization strains, had similar morphology and filamentous growth, which confirms that the formation of foci was not due to stress induced by overproduction of Tpk subunits. On the other hand, formation of similar distinct cytoplasmic foci was already observed for various protein kinases in *S. cerevisiae* (Shah *et al.* 2014). It is suggested that they serve as cellular compartments. Even though we did not observe the enrichment with the full-length C-terminal yEmVenus fusions, it is possible that a similar PKA signaling regulation also exists in *C. albicans*. Possibly this mechanism is specific for the PKA holoenzyme, and can therefore be visualized only by using the BiFC system, which stabilizes the Bcy1-Tpk holoenzyme.

This specific localization of the PKA interaction complex could also indicate subcellular compartmentalization of PKA, which has been shown to be important for proper cAMP-mediated signaling (Griffioen and Thevelein 2002). In many multicellular organisms, A-kinase anchor proteins are known for fine-tuning PKA activity by targeting inactive PKA holoenzymes to specific intracellular locations (Colledge and Scott 1999). However, proteins with similar function have not been identified so far in yeast. Hence, for more detailed interpretation of our observations, further investigation needs to be done, to identify the functional relevance of these foci in *C. albicans* and confirm the presence of possible adaptor proteins.

Although the BiFC assay proved to be useful for identification of PPI in *C. albicans*, the limitations of the tool need to be considered. To verify positive interactions, it is critical to have appropriate controls. This can be done by expressing one of the interacting proteins tagged with one fluorophore part and the other with the complementary part, or ideally abolishing the interaction domain, if it is known. In the latter case, it is important to determine the stability of the BiFC protein fusion lacking the interaction domain, as we demonstrated in the case of the Snf4-Kis1 interaction pair. An additional limitation of the BiFC complex formation is its irreversibility *in vitro*, which can limit assessment of dynamic interactions. However, this irreversible nature can aid in visualizing weak or transient interactions (Miller *et al.* 2015). One of the advantages of the BiFC assay is that it is suitable for genome-wide PPI analysis, as recently shown in a study of the SUMO interactome in *S. cerevisiae* (Sung *et al.* 2013). Similarly, the adaptation of BiFC plasmids for Gateway cloning could enable the use of the BiFC assay for genome-wide PPI studies using the recently established *Candida* ORFeome library (Carol Munro, University of Aberdeen, and Christophe d’Enfert, Institut Pasteur). Mating-competent NV- and/or CV- tagged collections of *C. albicans* strains could be crossed for high-throughput screening. In this case, only one set of plasmids for tagging would be feasible to use. Finally, we underscore the fact that there is no one ideal method for PPI, and validation of interactions by other methods is highly desirable, as shown in this study using the *Candida* two-hybrid method.

In conclusion, we have successfully established an additional tool for PPI studies in *C. albicans*, by creating an optimized set of vectors allowing the use of BiFC in this organism. We validated our BiFC assay by demonstrating three interaction complexes *in vivo*, which were confirmed by *Candida* two-hybrid results. As a major advantage, our BiFC system allowed the visualization of the subcellular localization of the interaction complex, creating immense potential for elucidating novel (spatial) properties of molecular mechanisms operating in this important pathogen.

## 

## Supplementary Material

Supplemental material is available online at www.g3journal.org/lookup/suppl/doi:10.1534/g3.117.300149/-/DC1.
