# Supplementary figures and images for: A Bimolecular Fluorescence Complementation Tool for Identification of Protein-Protein Interactions in Candida albicans
Source: G3 (Bethesda). 2017 Aug 31;7(10):3509–20. doi: 10.1534/g3.117.300149 (PMC5633398; doi:10.1534/g3.117.300149)

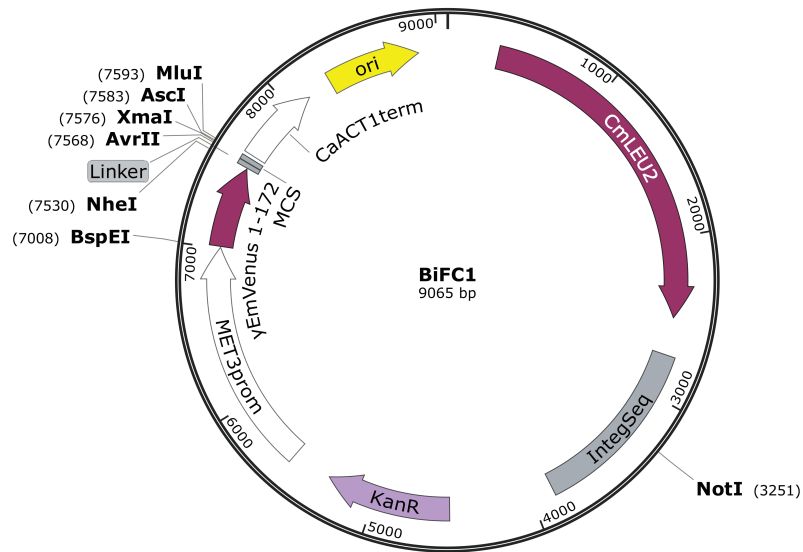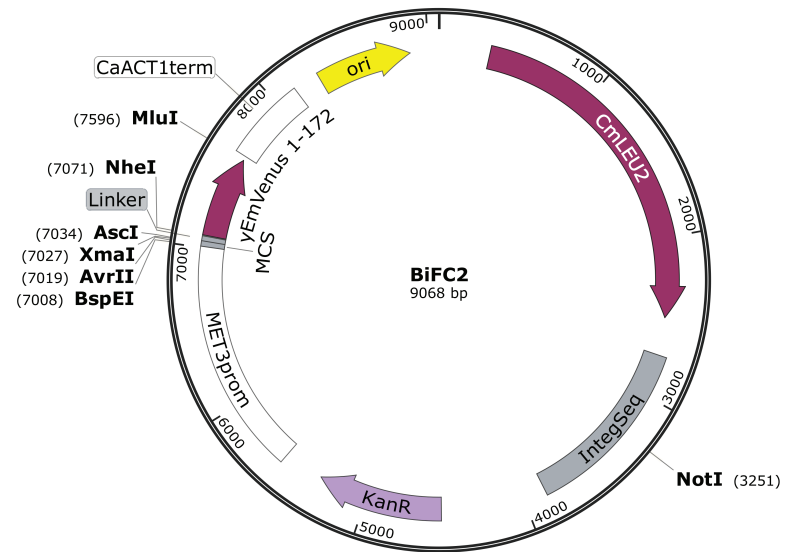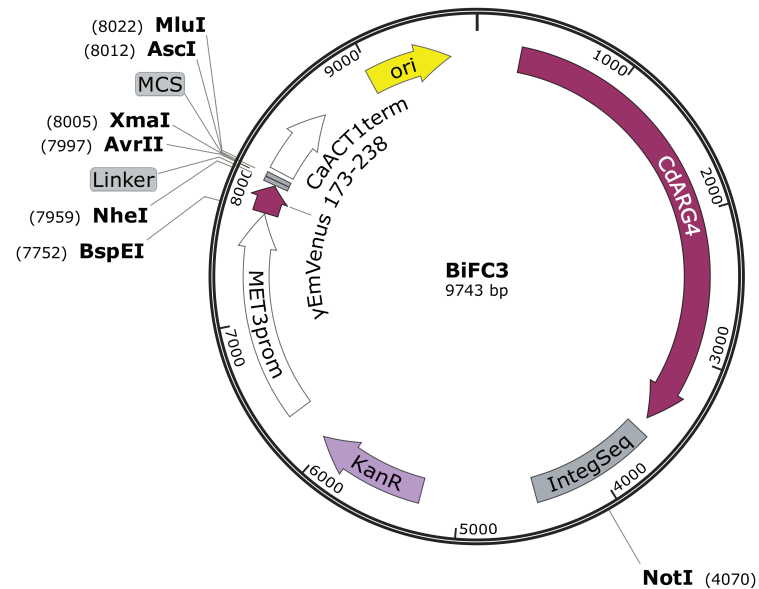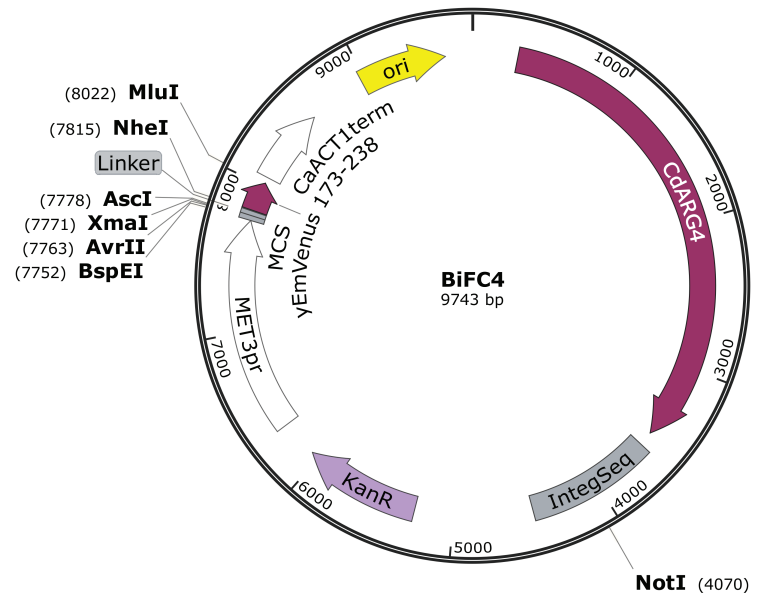

Supplement: Supplementary file 1 [file 3509FigureS1.pdf]
