# Supplementary material for: A Bimolecular Fluorescence Complementation Tool for Identification of Protein-Protein Interactions in Candida albicans
Source: G3 (Bethesda). 2017 Aug 31;7(10):3509–20. doi: 10.1534/g3.117.300149 (PMC5633398; doi:10.1534/g3.117.300149)
Supplement: Supplementary file 13 [file 3509FileS5.docx]

SUPPLEMENTARY FILES LEGENDS

File S1. Sequence of BiFC1 plasmid

File S2. Sequence of BiFC2 plasmid

File S3. Sequence of BiFC3 plasmid

File S4. Sequence of BiFC4 plasmid
